# Efficacy of extracellular vesicles of different cell origins in traumatic brain injury: A systematic review and network meta-analysis

**DOI:** 10.3389/fnins.2023.1147194

**Published:** 2023-03-29

**Authors:** Zhe-Lun Yang, Ze-Yan Liang, Yi-Ke Lin, Fa-Bin Lin, Jian Rao, Xiong-Jie Xu, Chun-Hua Wang, Chun-Mei Chen

**Affiliations:** Department of Neurosurgery, Fujian Medical University Union Hospital, Fuzhou, Fujian, China

**Keywords:** traumatic brain injury, network meta-analysis, extracellular vesicles, preclinical research, systematic review

## Abstract

**Background:**

There was still no effective treatment for traumatic brain injury (TBI). Recently, many preclinical studies had shown promising efficacy of extracellular vesicles (EVs) from various cell sources. Our aim was to compare which cell-derived EVs were most effective in treating TBI through a network meta-analysis.

**Methods:**

We searched four databases and screened various cell-derived EVs for use in preclinical studies of TBI treatment. A systematic review and network meta-analysis were conducted for two outcome indicators, modified Neurological Severity Score (mNSS) and Morris Water Maze (MWM), and they were ranked by the surface under the cumulative ranking curves (SUCRA). Bias risk assessment was performed with SYRCLE. R software (version 4.1.3, Boston, MA, USA) was used for data analysis.

**Results:**

A total of 20 studies were included in this study, involving 383 animals. Astrocyte-derived extracellular vesicles (AEVs) ranked first in response to mNSS at day 1 (SUCRA: 0.26%), day 3 (SUCRA: 16.32%), and day 7 (SUCRA: 9.64%) post-TBI. Extracellular vesicles derived from mesenchymal stem cells (MSCEVs) were most effective in mNSS assessment on day 14 (SUCRA: 21.94%) and day 28 (SUCRA: 6.26%), as well as MWM’s escape latency (SUCRA: 6.16%) and time spent in the target quadrant (SUCRA: 86.52%). The result of mNSS analysis on day 21 showed that neural stem cell-derived extracellular vesicles (NSCEVs) had the best curative effect (SUCRA: 6.76%).

**Conclusion:**

AEVs may be the best choice to improve early mNSS recovery after TBI. The efficacy of MSCEVs may be the best in the late mNSS and MWM after TBI.

**Systematic review registration:**

https://www.crd.york.ac.uk/prospero/, identifier CRD42023377350.

## 1. Introduction

Traumatic brain injury (TBI) is a kind of neurological or even motor dysfunction caused by brain trauma, which can be life-threatening in severe cases (Menon et al., 2010). In the epidemiological report of 2016, more than 8 million TBI patients will lead to Years Lived with Disability (YLDs), which brings a huge burden to society (Badhiwala et al., 2019). Therefore, the treatment of TBI has great significance for individuals, societies and countries.

To date, the recommended treatment for TBI includes hypertonic therapy, neurotrophic drug therapy, surgical decompression, and nutritional support (Maas et al., 2017, 2022). However, these methods do not work well. Therefore, new therapies are urgently needed to enhance the efficacy and improve the prognosis. As early as 20 years ago, there were studies showing that cell therapy has good promise (Mahmood et al., 2001; Chopp and Li, 2002). Therefore, a large number of studies have promoted the progress of cell therapy (Harvey and Chopp, 2003; Mahmood et al., 2004; Osanai et al., 2012). However, tumorigenicity and immune rejection are still troubling researchers when cell therapy is used in clinical studies (Liu et al., 2021).

Cell free therapy, an emerging therapy that is expected to replace cell therapy, has attracted a large number of researchers’ attention (Dutta et al., 2021; Pischiutta et al., 2022). The so-called cell-free therapy is the treatment of various diseases with products secreted by cells (also known as extracellular vesicles) (Han et al., 2022). Extracellular vesicle (EV) is a vesicle with membrane structure and rich contents produced by cells through endocytosis or exocytosis (van Niel et al., 2018). It has multiple subtypes and plays an irreplaceable role in the physiological and pathological processes of various diseases (Kalra et al., 2016). Specific information can be referred to the relevant review (Colombo et al., 2014; Thery et al., 2018).

In recent years, a large number of studies have shown that EVs can effectively treat various neurodegenerative diseases, including TBI (Guedes et al., 2020; Mot et al., 2022). There have also been conventional meta-analysis showing that EVs can improve neurological dysfunction after TBI (Muhammad et al., 2022). However, in the study of cell therapy, it has been found that the efficacy of different cells varies (Xu et al., 2022). This makes it all the more important to select a particular cell to treat a particular disease. To date, no studies have shown which cell-derived EVs work better. Therefore, the purpose of this study is to provide a cell source with the best curative effect by conducting a network meta-analysis of the efficacy of EVs from different cell sources in TBI, so as to provide ideas for preclinical research and promote the progress of cell-free therapies into clinical research.

## 2. Methods

### 2.1. Search strategy

The literature search was conducted in strict accordance with the expanded statement of the Preferred Reporting Items for Systematic Reviews and Meta-Analyses statement (PRISMA) (Hutton et al., 2015). The PROSPERO database registration number was CRD42023377350. A comprehensive search was conducted on PubMed, Ovid-Embase, The Cochrane Library, and Web of Science databases until November 10, 2022. The search strategy was based on English keywords and free words. Details could be found in Supplementary Table 1.

### 2.2. Study selection

Two researchers independently screened the study based on the inclusion and exclusion criteria established in strict accordance with the PICOS principle. Controversial studies were evaluated by a third investigator for resolution. Preclinical studies of EVs for the treatment of TBI were included regardless of the cellular origin of EVs. The outcome measures included modified Neurological Severity Score (mNSS) and Morris Water Maze (MWM) (Gold et al., 2013). The mNSS had been used to assess neural function after TBI, and MWM had been used to test learning and memory ability in animals. Exclusion criteria included: (1) *in vitro* and clinical studies; (2) Reviews, letters and comments; (3) There was no mNSS or MWM outcome indicator.

### 2.3. Data extraction and quality assessment

The data were extracted independently by two specialized researchers and examined by another trained researcher. A third researcher was also involved in discussing and resolving their conflicting data. Information extracted included authors, year, country, groups, sample size, animal species, sex, age, weight, type of model, EVs source, immunocompatibility, EVs separation method, time, dose, frequency and method of administration, and outcome indicators. The raw data for outcome metrics was extracted using GetData Graph Digitizer (version 2.24). Two evaluators independently assessed the quality of each included study using the Systematic Review Center for Laboratory Animal Experimentation (SYRCLE) bias risk assessment tool^1^ (Hooijmans et al., 2014). Review Manager 5.4.1 was used to draw bias risk maps and summaries.

### 2.4. Statistical analysis

The network meta-analysis based on the Bayesian approach was performed using R software (version 4.1.3, Boston, MA, USA) for statistical analysis (Jansen et al., 2008). The nodes in the network diagram represent the intervention and the control group, and the sample size is represented by the node size. The line between nodes represents a direct comparison between two nodes, and the number of studies of direct comparison can be seen in the width of the line. When Markov Chain Monte Carlo (MCMC) method was used, the annealing times were 20,000, and the iteration times were 50,000 (Gressani et al., 2022). The continuous variables were evaluated using mean difference (MD) and 95% confidence interval (95% CI), and the corresponding results were presented in the form of forest map. League tables were used to show the results of direct and indirect comparisons. The surface under the cumulative ranking curves (SUCRA) was used to evaluate the ranking of different interventions (Salanti et al., 2011). The tightly controlled inclusion and exclusion criteria make this network meta-analysis maintain good similarity (Salanti, 2012). The consistency of data can be judged by comparing the deviance information criterion (DIC) values of different models (Zhu et al., 2014). The smaller the difference value is, the higher the consistency is. By definition, there are no local inconsistencies in a closed-loop study (White et al., 2012). Heterogeneity between studies could be assessed by the I^2^ value. Non-statistically significant heterogeneity results showed an I^2^ value <50%. The publication bias was checked by using funnel plot and Egger’s regression test. Testing for publication bias was not required for meta-analyses with fewer than 10 studies (Stang, 2010).

## 3. Results

### 3.1. Study characteristics

**FIGURE 1:**
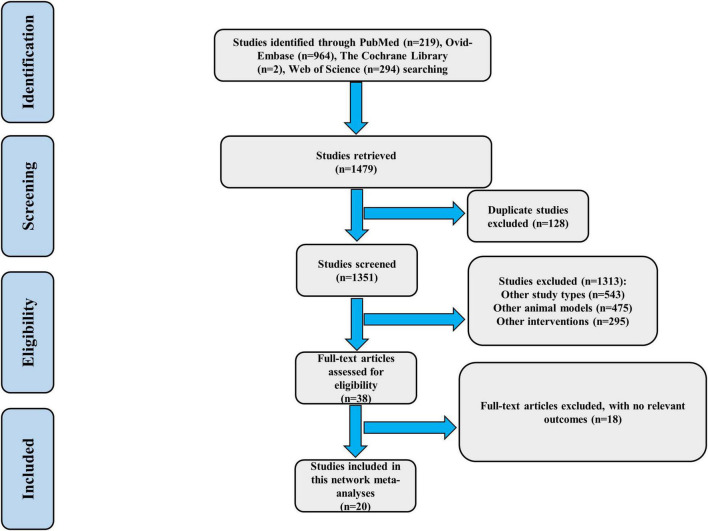
Flow chart of screening study.

A total of 1,479 studies were obtained through a comprehensive search of relevant databases. 128 duplicates were excluded. After preliminary screening of 1313 studies by title and abstract, 38 studies remained after excluding 1313 studies. After reading the full text, 18 studies without relevant outcome indicators were excluded. Finally, a total of 20 eligible studies were included in the network meta-analysis (Figure 1).

The included studies were published between 2015 and 2022, with three from the United States (15%), one from Iran (5%), and the rest all from China (80%). The study included a sample size of 383 animals. Seven of the studies involved mice (35%) and 13 involved rats (65%). All used male animals (90%), except for one study (5%) in which the animals were not gender-specific and one (5%) in which female rats were used. The ages ranged from 6 to 14 weeks, with mice weighing 20–25 g and rats weighing 200–352.8 g. The majority of the studies (80%) chose the CCI model, while a small percentage (10%) chose the WD model. Only one study chose the FPI model (5%). There’s even one study (5%) that didn’t exactly describe how it’s made. Cell sources for EVs were mesenchymal stem cell (MSC, 65%), astrocyte (20%), microglia (10%) and neural stem cell (NSC, 5%). Allogeneic (50%) and xenogeneic (50%) were used for EVs cell origin studies. The majority of studies (65%) used traditional ultracentrifugation methods to isolate EVs. Some (25%) used EVs isolation kits. EVs had also been obtained using ultrafiltration (5%) and density-gradient ultracentrifugation (5%) methods. Most studies (80%) gave the EVs within 1 day of injury. One study (5%) administered drugs 35 days after injury. Another study (5%) compared 1, 4, and 7 days of administration. However, there were also two studies (10%) that did not specify the timing of dosing. The majority of studies (85%) were dosed in a single dose of 3−200 μg. One study (5%) quantified EVs dose using particle count. Another study (5%) quantified the number of EVs-derived cells. Of course, one study (5%) did not specify dose or frequency. EVs was administered intravenously (65%), intraventricular (30%), and retroorbital (5%). Table 1 summarized the research characteristics. By comparing the DIC values of different models (Supplementary Table 2), it was found that the consistency of all outcome indexes was high.

**TABLE 1:** Characteristics of included studies.

References	Country	Groups (sample size)	Animal species	Sex	Age	Weight	Type of model	EVs source	Immunocompatibility	EVs separation method	Time, dose, frequency, and method of administration	Outcome indicators
Zhang et al., 2015	United States	1. TBI + EVs (*n* = 8) 2. TBI + PBS (*n* = 8) 3. Sham (*n* = 8)	Wistar rats	Male	2−3 months	325 ± 11 g	CCI	MSC	Allogeneic	Kit	1 day after TBI, 100 μg, 1 dose, and Intravenous injection	mNSS and MWM
Kim et al., 2016	United States	1. TBI + EVs (*n* = 10) 2. TBI + PBS (*n* = 15) 3. Sham (*n* = 10)	C57BL/6J mice	Male	7−8 weeks	Unclear	CCI	MSC	Xenogeneic	Ultracentrifugation	1 h after TBI, 30 μg, 1 dose, and Intravenous injection	MWM
Zhang et al., 2017	United States	1. TBI + EVs from MSCs in 3D culture (*n* = 8) 2. TBI + EVs from MSCs in 2D culture (*n* = 8) 3. TBI + liposomes (*n* = 8) 4. Sham (*n* = 8)	Wistar rats	Male	2−3 months	317 ± 10 g	CCI	MSC	Xenogeneic	Kit	1 day after TBI, 100 μg, 1 dose, and Intravenous injection	mNSS and MWM
Li et al., 2019	China	1. TBI + miR-124−3p-downregulated EVs (*n* = 8) 2. TBI + miR-124−3p-upregulated EVs (*n* = 8) 3. TBI + EVs (*n* = 8) 4. TBI + PBS (*n* = 8) 5. Sham (*n* = 8)	C57BL/6J mice	Male	10−12 weeks	20−25 g	CCI	Microglia	Allogeneic	Ultracentrifugation	1 h after TBI, 30 μg, 1 dose, and Intravenous injection	mNSS and MWM
Ni et al., 2019	China	1. TBI + EVs (*n* = 7) 2. TBI + PBS (*n* = 7) 3. Sham (*n* = 7)	C57BL/6J mice	Male	12−14 weeks	Unclear	CCI	MSC	Allogeneic	Ultracentrifugation	15 min after TBI, 30 μg, 1 dose, and Retro-orbital injection	mNSS
Wang and Han, 2019	China	1. TBI + EVs carrying pIRES2-EGFP-Bcl-2 plasmid and EGFP-C1-Bax shRNA plasmid (*n* = 15) 2. TBI + EVs (*n* = 15) 3. Sham (*n* = 15)	C57BL/6J mice	Unclear	Unclear	Unclear	CCI	Astrocyte	Allogeneic	Ultracentrifugation	1 h after TBI, 10 μg, 1 dose, and Intraventricular injection	MWM
Yang et al., 2019	China	1. TBI + miR-124 Enriched EVs (*n* = 6) 2. TBI (*n* = 6) 3. Sham + miR-124 Enriched EVs (*n* = 6) 4. Sham (*n* = 6)	SD rats	Male	8−10 weeks	250−300 g	CCI	MSC	Allogeneic	Kit	1 day after TBI, 100 μg, 1 dose, and Intravenous injection	NSS and MWM
Chen et al., 2020	China	1. TBI + MSC (*n* = 8) 2. TBI + EVs (*n* = 8) 3. TBI + PBS (*n* = 8) 4. Sham (*n* = 8)	SD rats	Male	6−8 weeks	300 ± 11 g	WD	MSC	Xenogeneic	Ultracentrifugation	1 day after TBI, 20 μg, 1 dose, and Intraventricular injection	mNSS
Ge et al., 2020	China	1. TBI + miR-124 enriched EVs (*n* = 10) 2. TBI + EVs (*n* = 10) 3. TBI (*n* = 10) 4. Sham (*n* = 10)	C57BL/6J mice	Male	12 weeks	20−25 g	CCI	Microglia	Allogeneic	Ultracentrifugation	35 days after TBI, 3 × 10^∧^10 particles, 1 dose, and Intravenous injection	MWM
Liu et al., 2020	China	1. TBI + EVs by injury brain extract (*n* = 12) 2. TBI + EVs (*n* = 12) 3. TBI (*n* = 12) 4. Sham (*n* = 12)	SD rats	Male	8 weeks	250−300 g	FPI	MSC	Xenogeneic	Ultrafiltration	Immediately after TBI, EVs releasing from 1.5 × 10^∧^6 cells, 1 dose, and Intraventricular injection	MWM
Long et al., 2020	China	1. TBI + miR-873a-5p enriched EVs (*n* = 5) 2. TBI (*n* = 5) 3. Sham + miR-873a-5p enriched EVs (*n* = 5) 4. Sham (*n* = 5)	C57BL/10ScNJ mice	Male	10−12 weeks	20−22 g	CCI	Astrocyte	Allogeneic	Ultracentrifugation	20 min after TBI, Unclear, and Intraventricular injection	mNSS
Xu et al., 2020	China	1. TBI + BDNF-induced EVs (*n* = 6) 2. TBI + EVs (*n* = 6) 3. TBI + PBS (*n* = 6) 4. Sham (*n* = 6)	SD rats	Male	Unclear	220−250 g	CCI	MSC	Allogeneic	Ultracentrifugation	1 day after TBI, 100 μg, 1 dose, and Intravenous injection	mNSS and MWM
Zhang et al., 2020	China	1. TBI + 200 μg EVs (*n* = 8) 2. TBI + 100 μg EVs (*n* = 8) 3. TBI + 50 μg EVs (*n* = 8) 4. TBI + PBS (*n* = 8) 5. Sham (*n* = 8)	Wistar rats	Male	3 months	339.2 ± 13.6 g	CCI	MSC	Xenogeneic	Ultracentrifugation	1, 4, or 7 days after TBI, 50, 100, or 200 μg, 1 dose, and Intravenous injection	mNSS and MWM
He et al., 2021	China	1. TBI + EVs NKILA (*n* = 15) 2. TBI + EVs (*n* = 15) 3. TBI (*n* = 15) 4. Sham (*n* = 15)	C57BL/10ScNJ mice	Male	10−12 weeks	20−22 g	CCI	Astrocyte	Xenogeneic	Ultracentrifugation	Unclear, 3 ± 0.75 μg, 1 dose, and Intraventricular injection	mNSS
Zhang W. et al., 2021	China	1. TBI + EVs (*n* = 8) 2. TBI (*n* = 8) 3. Sham + EVs (*n* = 8) 4. Sham (*n* = 8)	SD rats	Male	Unclear	210−260 g	CCI	Astrocyte	Allogeneic	Ultracentrifugation	30 min after TBI, 100 μg, 1 dose, and Intravenous injection	mNSS and MWM
Zhang Y. et al., 2021	China	1. TBI + miR-17−92 enriched EVs (*n* = 8) 2. TBI + EVs (*n* = 8) 3. TBI + PBS (*n* = 8) 4. Sham (*n* = 8)	Wistar rats	Male	2−3 months	Unclear	CCI	MSC	Xenogeneic	Ultracentrifugation	1 day after TBI, 100 μg, 1 dose, and Intravenous injection	mNSS and MWM
Abedi et al., 2022	Iran	1. TBI + NSC (*n* = 10) 2. TBI + EVs (*n* = 10) 3. TBI (*n* = 10)	Wistar rats	Male	Unclear	220−250 g	Unclear	NSC	Xenogeneic	Kit	Unclear, 63 μg, 1 dose, and Intraventricular injection	mNSS
Cui et al., 2022	China	1. TBI + EVs (*n* = 15) 2. TBI + PBS (*n* = 15) 3. Sham (*n* = 15)	SD rats	Female	Unclear	200−220 g	WD	MSC	Xenogeneic	Ultracentrifugation	1 day after TBI, 200 μg, 1 dose, and Intravenous injection	mNSS
Zhang et al., 2022	China	1. TBI + EVs (*n* = 15) 2. TBI + PBS (*n* = 15) 3. Sham (*n* = 15)	SD rats	Male	Unclear	250−300 g	CCI	MSC	Xenogeneic	Density-gradient ultracentrifugation	1 day after TBI, 100 μg, 1 dose, and Intravenous injection	mNSS and MWM
Zhuang et al., 2022	China	1. TBI + SB203580 (*n* = 7) 2. TBI + EVs (*n* = 7) 3. TBI + PBS (*n* = 7) 4. Sham (*n* = 7)	SD rats	Male	6−8 weeks	250 ± 50 g	CCI	MSC	Allogeneic	Kit	1 h after TBI, 100 μg, 1 dose, and Intravenous injection	MWM

TBI, traumatic brain injury; EVs, extracellular vesicles; CCI, controlled cortical impact; FPI, fluid percussion injury; WD, weight-drop; MSC, mesenchymal stem cell; NSC, neural stem cell; SD, Sprague-Dawley; MWM, Morris Water Maze; mNSS, modified Neurological Severity Score; PBS, phosphate buffer saline; EGFP, enhanced green fluorescent protein; BDNF, brain-derived neurotrophic factor; NKILA, nuclear factor-κB interacting lncRNA p38 MAPK inhibitor SB203580.

### 3.2. Comparison of the efficacy of EVs from different cell sources in mNSS at day 1 post-TBI

**FIGURE 2:**
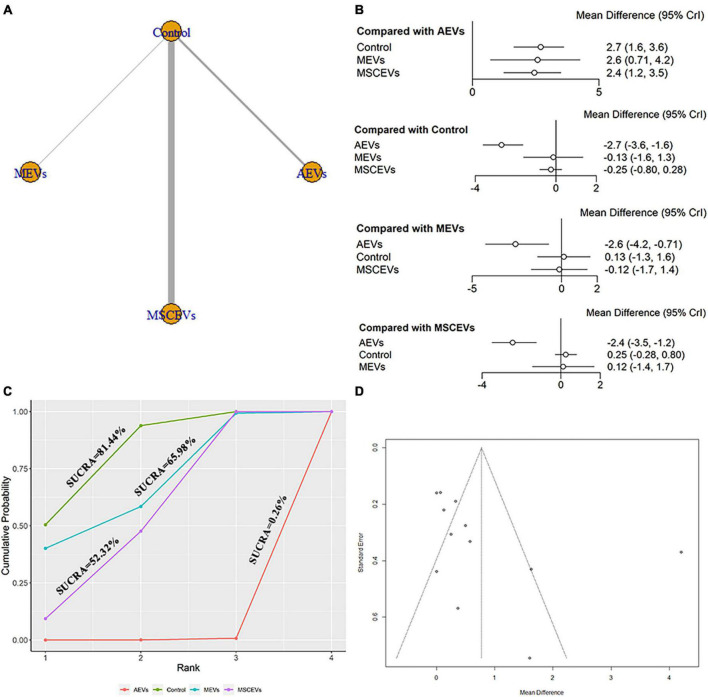
Comparison of the efficacy of extracellular vesicles (EVs) from different cell sources in modified Neurological Severity Score (mNSS) at day 1 post-traumatic brain injury (TBI). **(A)** Network plot; **(B)** Forest plot; **(C)** Surface under the cumulative ranking curves (SUCRA); **(D)** Funnel plot. The dotted lines represent 95% confidence interval (95% CI). AEVs, astrocyte-derived extracellular vesicles; MEVs, microglia-derived extracellular vesicles; MSCEVs, mesenchymal stem cell-derived extracellular vesicles; NSCEVs, neural stem cell-derived extracellular vesicles.

The mNSS neurological function assessment at day 1 after TBI, a total of 12 studies were eligible (Zhang et al., 2015, 2017, 2020; Li et al., 2019; Ni et al., 2019; Chen et al., 2020; Long et al., 2020; Xu et al., 2020; He et al., 2021; Zhang W. et al., 2021; Zhang Y. et al., 2021; Cui et al., 2022). The network diagram (Figure 2A) showed three interventions and control comparisons, including astrocyte-derived extracellular vesicles (AEVs), microglia-derived extracellular vesicles (MEVs) and mesenchymal stem cell-derived extracellular vesicles (MSCEVs). The results of forest map (Figure 2B) showed that, compared with control [MD: −2.7, 95%CI: (−3.6, −1.6)], MEVs [MD: −2.6, 95%CI: (−4.2, −0.71)] and MSCEVs [MD: −2.4, 95%CI: (−3.5, −1.2)], AEVs were more effective. The result of SUCRA (Figure 2C) showed that AEVs (0.26%) had the best curative effect, followed by MSCEVs (52.32%), MEVs (65.98%) and Control (81.44%). For mNSS, the lower the score, the better the recovery of neural function. Therefore, the closer the SUCRA value is to 0%, the better the efficacy is. The funnel plot (Figure 2D) and Egger’s test (Table 2, *p*-value = 0.0986) showed that publication bias did not exist.

**TABLE 2:** Egger’s test of relevant outcome indicators.

Linear regression test of funnel plot asymmetry				
mNSS-D1	Test result: *t* = 1.82, df = 10, *p*-value = 0.0986			
	bias	se.bias	intercept	se.intercept
	3.978	2.1844	−0.529	0.5582
mNSS-D7	Test result: *t* = −1.82, df = 13, *p*-value = 0.0926			
	bias	se.bias	intercept	se.intercept
	−3.7083	2.043	4.1243	0.7703
mNSS-D14	Test result: *t* = −2.48, df = 12, *p*-value = 0.0289			
	bias	se.bias	intercept	se.intercept
	−4.8067	1.9369	4.1811	0.58
mNSS-D21	Test result: *t* = −1.03, df = 8, *p*-value = 0.3315			
	bias	se.bias	intercept	se.intercept
	−3.7254	3.6037	4.2349	1.0319
MWM-escape latency	Test result: *t* = 1.09, df = 11, *p*-value = 0.2978			
	bias	se.bias	intercept	se.intercept
	3.7029	3.3883	5.1648	5.6664
MWM-time spent in the goal quadrant	Test result: *t* = −1.90, df = 12, *p*-value = 0.0820			
	bias	se.bias	intercept	se.intercept
	−6.5814	3.4669	−0.1414	3.4923

### 3.3. Comparison of the efficacy of EVs from different cell sources in mNSS at day 3 post-TBI

**FIGURE 3:**
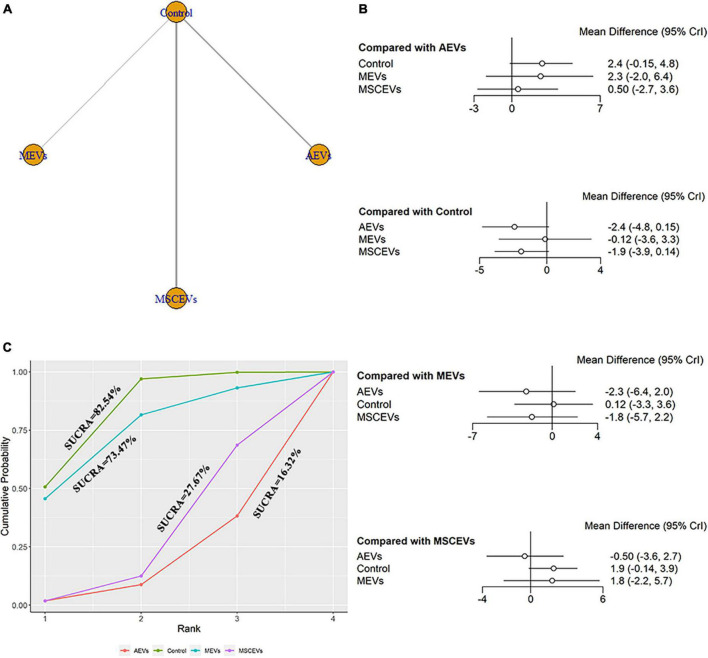
Comparison of the efficacy of extracellular vesicles (EVs) from different cell sources in modified Neurological Severity Score (mNSS) at day 3 post-traumatic brain injury (TBI). **(A)** Network plot; **(B)** Forest plot; **(C)** Surface under the cumulative ranking curves (SUCRA). AEVs, astrocyte-derived extracellular vesicles; MEVs, microglia-derived extracellular vesicles; MSCEVs, mesenchymal stem cell-derived extracellular vesicles; NSCEVs, neural stem cell-derived extracellular vesicles.

Six studies qualified for the mNSS neurological function assessment at day 3 after TBI (Li et al., 2019; Ni et al., 2019; Chen et al., 2020; Long et al., 2020; He et al., 2021; Cui et al., 2022). The network diagram (Figure 3A) showed three interventions and control comparisons, including AEVs, MEVs, and MSCEVs. No statistically significant comparison was found between the results of forest map (Figure 3B). The result of SUCRA (Figure 3C) showed that AEVs (16.32%) had the best curative effect, followed by MSCEVs (27.67%), MEVs (73.47%), and Control (82.54%).

### 3.4. Comparison of the efficacy of EVs from different cell sources in mNSS at day 7 post-TBI

**FIGURE 4:**
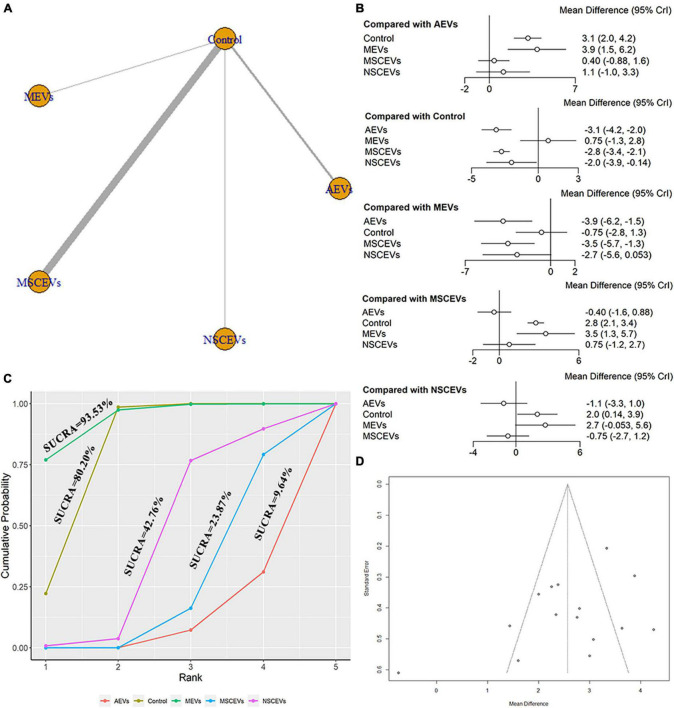
Comparison of the efficacy of extracellular vesicles (EVs) from different cell sources in mNSS at day 7 post-traumatic brain injury (TBI). **(A)** Network plot; **(B)** Forest plot; **(C)** Surface under the cumulative ranking curves (SUCRA); **(D)** Funnel plot. The dotted lines represent 95% confidence interval (95% CI). AEVs, astrocyte-derived extracellular vesicles; MEVs, microglia-derived extracellular vesicles; MSCEVs, mesenchymal stem cell-derived extracellular vesicles; NSCEVs, neural stem cell-derived extracellular vesicles.

The mNSS neurological function assessment at day 7 after TBI, a total of 15 studies were eligible (Zhang et al., 2015, 2017, 2020; Li et al., 2019; Ni et al., 2019; Yang et al., 2019; Chen et al., 2020; Long et al., 2020; Xu et al., 2020; He et al., 2021; Zhang W. et al., 2021; Zhang Y. et al., 2021; Abedi et al., 2022; Cui et al., 2022; Zhang et al., 2022). The network diagram (Figure 4A) showed four interventions and control comparisons, including AEVs, MEVs, neural stem cell-derived extracellular vesicles (NSCEVs) and MSCEVs. The results of forest map (Figure 4B) showed that compared with the control, AEVs [MD: −3.1, 95% CI: (−4.2, −2.0)], MSCEVs [MD: −2.8, 95% CI: (−3.4, −2.1)] and NSCEVs [MD: −2.0, 95% CI: (−3.9, −0.14)] could effectively improve the neurological function after TBI. Compared with MEVs, AEVs [MD: −3.9, 95% CI: (−6.2, −1.5)] and MSCEVs [MD: −3.5, 95% CI: (−5.7, −1.3)] were more beneficial to neurological recovery after TBI. The result of SUCRA (Figure 4C) showed that AEVs (9.64%) had the best curative effect, followed by MSCEVs (23.87%), NSCEVs (42.76%), Control (80.2%), and MEVs (93.53%). The funnel plot (Figure 4D) and Egger’s test (Table 2, *p*-value = 0.0926) showed that publication bias did not exist.

### 3.5. Comparison of the efficacy of EVs from different cell sources in mNSS at day 14 post-TBI

**FIGURE 5:**
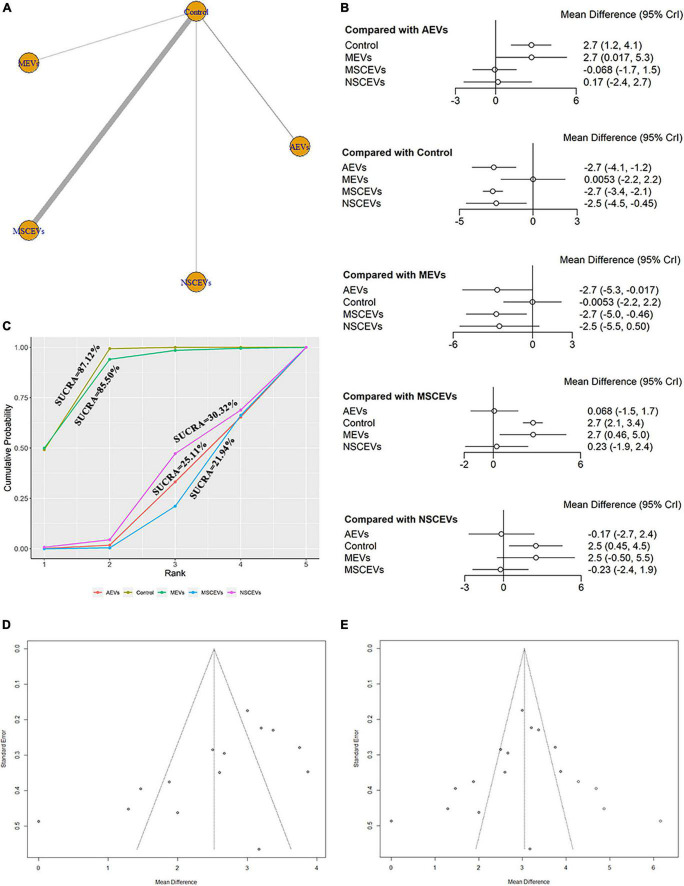
Comparison of the efficacy of extracellular vesicles (EVs) from different cell sources in modified Neurological Severity Score (mNSS) at day 14 post-traumatic brain injury (TBI). **(A)** Network plot; **(B)** Forest plot; **(C)** Surface under the cumulative ranking curves (SUCRA); **(D)** Funnel plot; **(E)** Funnel plot by the trim and fill analysis method. The dotted lines represent 95% confidence interval (95% CI). AEVs, astrocyte-derived extracellular vesicles; MEVs, microglia-derived extracellular vesicles; MSCEVs, mesenchymal stem cell-derived extracellular vesicles; NSCEVs, neural stem cell-derived extracellular vesicles.

The mNSS neurological function assessment at day 14 after TBI, a total of 14 studies were eligible (Zhang et al., 2015, 2017, 2020; Li et al., 2019; Ni et al., 2019; Yang et al., 2019; Chen et al., 2020; Long et al., 2020; Xu et al., 2020; He et al., 2021; Zhang Y. et al., 2021; Abedi et al., 2022; Cui et al., 2022; Zhang et al., 2022). The network diagram (Figure 5A) showed four interventions and control comparisons, including AEVs, MEVs, NSCEVs, and MSCEVs. The results of forest map (Figure 5B) showed that, compared with the control, AEVs [MD: −2.7, 95% CI: (−4.1, −1.2)], MSCEVs [MD: −2.7, 95% CI: (−3.4, −2.1)] and NSCEVs [MD: −2.5, 95% CI: (−4.5, −0.45)] could effectively improve the neurological function after TBI. Compared with MEVs, AEVs [MD: −2.7, 95% CI: (−5.3, −0.017)] and MSCEVs [MD: −2.7, 95% CI: (−5.0, −0.46)] significantly improved mNSS neural function scores. The results of SUCRA (Figure 5C) showed that MSCEVs (21.94%) had the best curative effect, followed by AEVs (25.11%), NSCEVs (30.32%), MEVs (85.5%), and Control (87.12%). The results of funnel plot (Figure 5D) and Egger’s test (Table 2, *p*-value = 0.0289) indicated that publication bias might exist. Therefore, we found four “missing” studies on the right side of the funnel plot by the trim and fill analysis method (Figure 5E).

### 3.6. Comparison of the efficacy of EVs from different cell sources in mNSS at day 21 post-TBI

**FIGURE 6:**
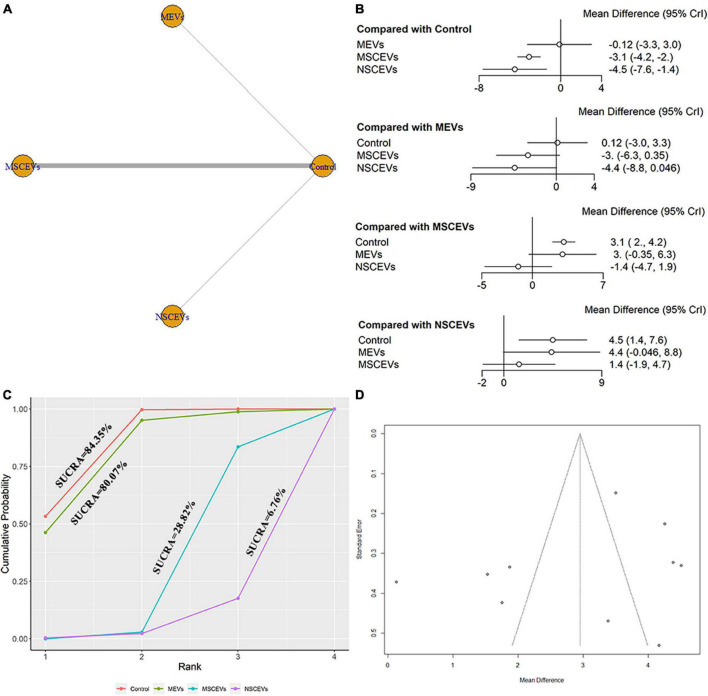
Comparison of the efficacy of extracellular vesicles (EVs) from different cell sources in modified Neurological Severity Score (mNSS) at day 21 post-traumatic brain injury (TBI). **(A)** Network plot; **(B)** Forest plot; **(C)** Surface under the cumulative ranking curves (SUCRA); **(D)** Funnel plot. The dotted lines represent 95% confidence interval (95% CI). AEVs, astrocyte-derived extracellular vesicles; MEVs, microglia-derived extracellular vesicles; MSCEVs, mesenchymal stem cell-derived extracellular vesicles; NSCEVs, neural stem cell-derived extracellular vesicles.

The mNSS neurological function assessment at day 21 after TBI, a total of 10 studies were eligible (Zhang et al., 2015, 2017, 2020; Li et al., 2019; Yang et al., 2019; Chen et al., 2020; Zhang Y. et al., 2021; Abedi et al., 2022; Cui et al., 2022; Zhang et al., 2022). The network diagram (Figure 6A) showed three interventions and control comparisons, including MEVs, NSCEVs, and MSCEVs. The results of forest map (Figure 6B) showed that MSCEVs [MD: −3.1, 95% CI: (−4.2, −2.0)] and NSCEVs [MD: −4.5, 95% CI: (−7.6, −1.4)] could effectively improve the neurological function after TBI compared with the control. The results of SUCRA (Figure 6C) showed that NSCEVs (6.76%) had the best curative effect, followed by MSCEVs (28.82%), MEVs (80.07%), and Control (84.35%). The funnel plot (Figure 6D) and Egger’s test (Table 2, *p*-value = 0.3315) showed that publication bias did not exist.

### 3.7. Comparison of the efficacy of EVs from different cell sources in mNSS at day 28 post-TBI

**FIGURE 7:**
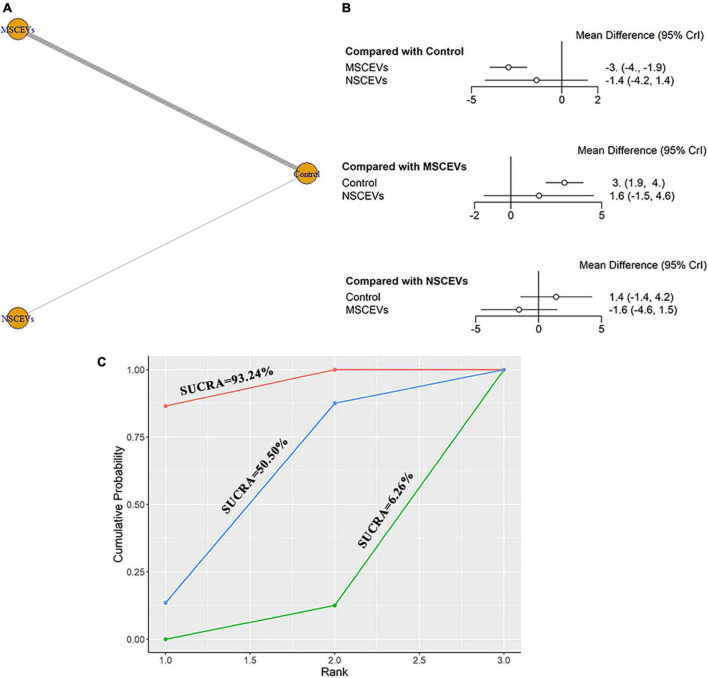
Comparison of the efficacy of extracellular vesicles (EVs) from different cell sources in modified Neurological Severity Score (mNSS) at day 28 post- traumatic brain injury (TBI). **(A)** Network plot; **(B)** Forest plot; **(C)** Surface under the cumulative ranking curves (SUCRA). AEVs, astrocyte-derived extracellular vesicles; MEVs, microglia-derived extracellular vesicles; MSCEVs, mesenchymal stem cell-derived extracellular vesicles; NSCEVs, neural stem cell-derived extracellular vesicles.

A total of 8 studies were eligible for mNSS neurological function assessment at day 28 after TBI (Zhang et al., 2015, 2017, 2020; Yang et al., 2019; Chen et al., 2020; Zhang Y. et al., 2021; Abedi et al., 2022; Zhang et al., 2022). The network diagram (Figure 7A) showed two interventions and control comparisons, including NSCEVs and MSCEVs. The results of forest map (Figure 7B) showed that MSCEVs [MD: −3.0, 95% CI: (−4.0, −1.9)] had significant curative effect compared with the control. The results of SUCRA (Figure 7C) showed that MSCEVs (6.26%) had the best curative effect, followed by NSCEVs (50.5%) and Control (93.24%).

### 3.8. Comparison of the efficacy of EVs from different cell sources in escape latency of MWM

**FIGURE 8:**
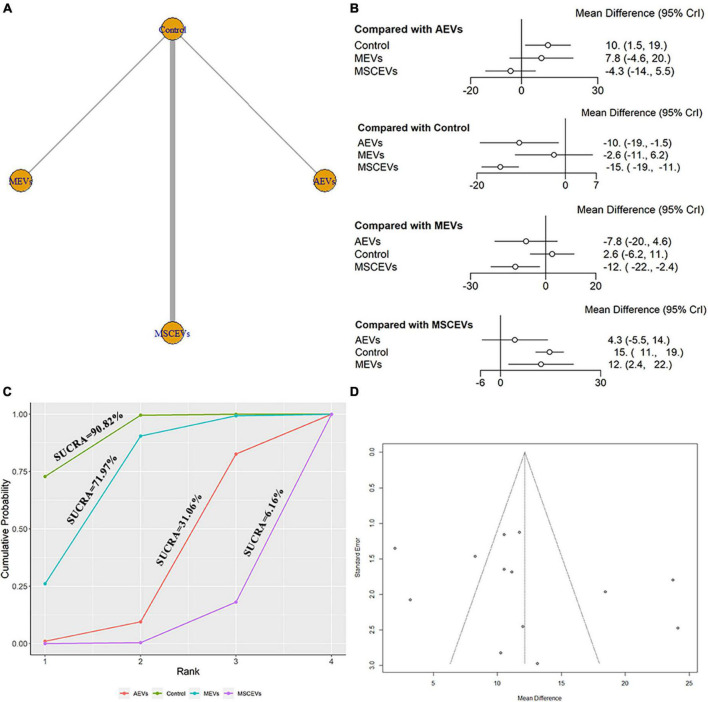
Comparison of the efficacy of extracellular vesicles (EVs) from different cell sources in escape latency of Morris Water Maze (MWM). **(A)** Network plot; **(B)** Forest plot; **(C)** Surface under the cumulative ranking curves (SUCRA); **(D)** Funnel plot. The dotted lines represent 95% confidence interval (95% CI). AEVs, astrocyte-derived extracellular vesicles; MEVs, microglia-derived extracellular vesicles; MSCEVs, mesenchymal stem cell-derived extracellular vesicles; NSCEVs, neural stem cell-derived extracellular vesicles.

We extracted data from the last day of escape latency in MWM for evaluation. A total of 13 studies were included in the network meta-analysis (Kim et al., 2016; Zhang et al., 2017, 2020; Li et al., 2019; Wang and Han, 2019; Yang et al., 2019; Ge et al., 2020; Liu et al., 2020; Xu et al., 2020; Zhang W. et al., 2021; Zhang Y. et al., 2021; Zhang et al., 2022; Zhuang et al., 2022). The network diagram (Figure 8A) showed three interventions and control comparisons, including AEVs, MEVs, and MSCEVs. The results of forest map (Figure 8B) showed that AEVs [MD: −10.0, 95% CI: (−19.0, −1.5)] and MSCEVs [MD: −15.0, 95% CI: (−19.0, −11.0)] reduced the escape latency time more than the control group, indicating that they could effectively improve the spatial memory ability after TBI. Compared with MEVs, MSCEVs [MD: −12.0, 95% CI: (−22.0, −2.4)] showed better efficacy. The shorter the escape latency time is, the better the spatial memory ability is. Therefore, the closer the SUCRA value is to 0%, the better the efficacy is. The results of SUCRA (Figure 8C) showed that MSCEVs (6.16%) had the best curative effect, followed by AEVs (31.06%), MEVs (71.97%), and Control (90.82%). The funnel plot (Figure 8D) and Egger’s test (Table 2, *p*-value = 0.2978) showed that publication bias did not exist.

### 3.9. Comparison of the efficacy of EVs from different cell sources in time spent in the goal quadrant in MWM

**FIGURE 9:**
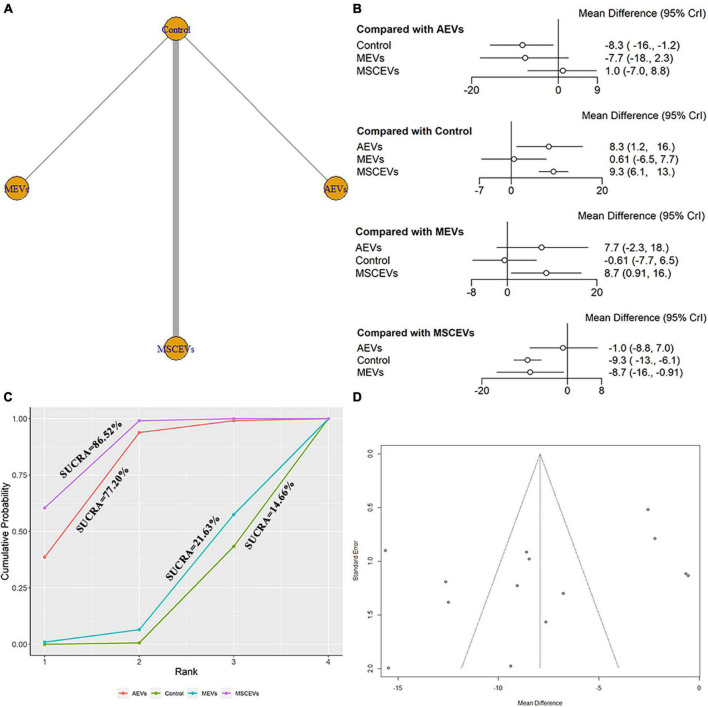
Comparison of the efficacy of extracellular vesicles (EVs) from different cell sources in time spent in the goal quadrant in Morris Water Maze (MWM). **(A)** Network plot; **(B)** Forest plot; **(C)** Surface under the cumulative ranking curves (SUCRA); **(D)** Funnel plot. The dotted lines represent 95% confidence interval (95% CI). AEVs, astrocyte-derived extracellular vesicles; MEVs, microglia-derived extracellular vesicles; MSCEVs, mesenchymal stem cell-derived extracellular vesicles; NSCEVs, neural stem cell-derived extracellular vesicles.

We extracted data from the last day of time spent in the goal quadrant in MWM for evaluation. A total of 14 studies were included in the network meta-analysis (Zhang et al., 2015, 2017, 2020; Kim et al., 2016; Li et al., 2019; Wang and Han, 2019; Yang et al., 2019; Ge et al., 2020; Liu et al., 2020; Xu et al., 2020; Zhang W. et al., 2021; Zhang Y. et al., 2021; Zhang et al., 2022; Zhuang et al., 2022). The network diagram (Figure 9A) shows three interventions and control comparisons, including AEVs, MEVs and MSCEVs. The results of forest map (Figure 9B) showed that AEVs [MD: 8.3, 95% CI: (1.2, 16.0)] and MSCEVs [MD: 9.3, 95% CI: (6.1, 13.0)] spent more time in the target quadrant than the control group, indicating that they could effectively improve the spatial memory ability after TBI. Compared with MEVs, MSCEVs [MD: 8.7, 95% CI: (0.91, 16.0)] showed better efficacy. The longer the time spent in the target quadrant, the better the spatial memory. Therefore, the closer the SUCRA value is to 100%, the better the efficacy is. The results of SUCRA (Figure 9C) showed that MSCEVs (86.52%) had the best curative effect, followed by AEVs (77.2%), MEVs (21.63%), and Control (14.65%). The funnel plot (Figure 9D) and Egger’s test (Table 2, *p*-value = 0.0820) showed that publication bias did not exist.

### 3.10. Risk of bias assessment

The results of the risk of bias assessment in this study were shown in Supplementary Figures 1A, B. Only two studies (10%) did not account for random sequence generation. About 50% of the studies were low risk for selection bias, performance bias, and detection bias. However, for all the included studies, it was not clear whether they processed incomplete outcome data. Therefore, all studies had the unclear risk of attrition bias. Moreover, only one study (5%) reported low risk, and the rest (95%) were unclear. For other bias, nine studies (45%) showed low risk of bias and five studies (25%) showed unclear risk of bias. However, there was a high risk of bias in six studies (30%). All in all, the included studies were of average quality.

## 4. Discussion

A total of 20 studies and 383 animals were included in this network meta-analysis. Our results suggested that AEVs, MEVs, NSCEVs, and MSCEVs were all effective in mNSS at day 1, 3, and 7 after TBI. Among them, the efficacy of AEVs was the most significant. Astrocytes are the most abundant glial cells in the central nervous system, and they play a crucial role in many neurodegenerative diseases (Hara et al., 2017; Escartin et al., 2021). After TBI, they play a role in maintaining the homeostasis of the internal environment and transmitting signals and communication between cells (Li et al., 2022; Yuan and Wu, 2022). In recent years, EV has been widely studied as a popular intercellular communication tool. It contains lipids, proteins, RNA and DNA and is responsible for its functional diversity (Colombo et al., 2014; Thery et al., 2018). A large number of studies have also shown that AEVs can be used to treat TBI, which can not only reduce the lesion volume, but also reduce cell apoptosis (Wang and Han, 2019; Long et al., 2020). In the early stages of TBI, astrocytes play an important role, which explains why AEVs are the most effective (Perez et al., 2017). However, due to the non-renewable nature of astrocytes, with the aggravation of secondary damage, the efficacy of AEVs diminishes with its depletion (Ridet et al., 1997; Sofroniew and Vinters, 2010). Therefore, it is not surprising that the efficacy of AEVs did not rank first in the late stage of TBI. However, NSCEVs ranked first in mNSS at day 21 after TBI. This result should be considered with caution as only one of the included studies used NSCEVs. Although studies have shown that NSCEVs can enhance the function of endogenous NSC after TBI, thus contributing to the recovery of neural function (Sun et al., 2020). However, the result of network meta-analysis showed NSCEVs were statistically significant compared with the control group, and there was no statistical difference between direct and indirect comparisons with other interventions in mNSS at day 21 after TBI. Therefore, more studies are needed to prove the efficacy of NSCEVs.

Moreover, AEVs, MEVs, NSCEVs, and MSCEVs were equally effective in mNSS at day 14 and 28 after TBI. Among them, MSCEVs ranked first in efficacy. As for the results of the network meta-analysis of the MWM test, AEVs, MEVs, and MSCEVs all had some effect. Among them, MSCEVs ranked first in the outcome analysis of escape latency and spent time in the target quadrant. As is well-known, MSC is a pluripotent stem cell with differentiation ability and therapeutic potential, which has been widely studied in various fields (Das et al., 2019; Shao et al., 2019). Stem cell therapies have also been used in clinical trials (Shahror et al., 2020). The efficacy of MSC in neurodegenerative diseases has also been widely reported (Peruzzaro et al., 2019; Köhli et al., 2021). However, although MSC has also been found to improve neurological function after TBI, inhibit the expression of apoptosis-related proteins Bax and Caspase−3, and also inhibit the secretion of pro-inflammatory and anti-inflammatory factors (Zhang et al., 2013; Shahror et al., 2019). Although MSC is easy to isolate and has homing function (Hsuan et al., 2016). However, it still has some unavoidable shortcomings such as tumorigenicity, easy contamination and immune rejection (Liu et al., 2021). Luckily, the discovery of MSCEVs is a bright spot. After TBI, the use of MSCEVs significantly improved the motor function and spatial memory ability of TBI animals (Patel et al., 2018; Moss et al., 2021). At the cellular level, MSCEVs can not only reduce apoptosis and neuroinflammatory response, but also promote the growth of progenies (Wen et al., 2022). One study has also shown that MSCEVs play a neuroprotective role by regulating the interaction between astrocytes and neurons through the PI3K/AKT signaling pathway (Turovsky et al., 2022). MSC contributes to the functional recovery of various endogenous cells and plays a more indirect role through continuous retention in the body (Andrzejewska et al., 2021). It also has the potential to differentiate into various cells, helping to replenish the cells consumed after TBI, thus exerting its curative effect. Therefore, it is natural that its efficacy ranked first in the late evaluation of mNSS and MWM. For another MEVs with therapeutic potential, it has been found to be useful in the treatment of early TBI. And they can also significantly inhibit inflammation. As resident macrophages of the central nervous system, microglia play an important role in the early stage of injury (Liddelow et al., 2017). The early inflammatory cascade reaction will affect axon regeneration, and microglia will also produce two polarization states of M1 and M2, affecting the secretion of inflammatory factors (Kigerl et al., 2009). One study has shown that EVs derived from microglia can promote axon regeneration through overexpression of miRNA, thus improving motor function after TBI (Zhao et al., 2021). Although MEVs were statistically significant compared to controls, they were less effective than other cell-derived EVs. It is possible that the intense early inflammatory response requires a large number of microglia, which are in short supply. It is also possible that some MEVs are affected by M1 microglia and turn into EVs that are not effective, thus playing a harmful role. Therefore, more studies are needed to further support the conclusion that MEVs can be used in the treatment of TBI.

Our Bayesian network meta-analysis provided a basis for future research by comparing EVs from different cell sources for TBI to select the EVs with the most significant efficacy. Our study not only assessed neural function, but also analyzed spatial memory ability. It is helpful for researchers to choose EVs which is more suitable for their research direction. To explore the mechanism of early efficacy, the use of MEVs and AEVs as intervention may be a better choice. MSCEVs and NSCEVs may be the best solution for researchers who want to further study the mechanism of neural function recovery after injury, or who want to compare the efficacy of stem cells and EVs. While providing strong evidence for preclinical research, it can reduce the detours in the transformation to clinical research.

There were some limitations to our Bayesian network meta-analysis. First, the number of studies included and the sample size were too small. The number of studies on some outcome measures was so small that some interventions had only been studied in one study, compromising the confidence of the results. Secondly, the quality of the included studies was uneven, and there were still studies with a high risk of bias. This might have something to do with the types of studies included. The unsatisfactory quality of preclinical research was still common due to the lack of systematic guidelines and inconsistent laboratory conditions. Moreover, the outcome indexes were all subjective results. The two outcome indicators, mNSS and MWM, were not objective enough, which might increase the risk of bias. Finally, SUCRA results used to assess efficacy rankings might have their own limitations affecting the results.

## 5. Conclusion

The results of our network meta-analysis suggested that AEVs might be the best option in the treatment of early TBI in mNSS. In the later stages of TBI, MSCEVs might have the best efficacy, both in neural function and spatial memory. However, more research is needed to confirm our findings and provide sufficient basis for its transformation into clinical research.

## Data availability statement

The raw data supporting the conclusions of this article will be made available by the authors, without undue reservation.

## Author contributions

Z-LY and Z-YL developed the ideas, wrote the frameworks and full texts, searched the literatures, and collected the data. Y-KL and F-BL analyzed and organized the data. JR and X-JX formatted the pictures and tables. C-HW and C-MC revised the article and reviewed the final version. All authors contributed to the article and approved the submitted version.

## Footnotes

1
http://www.biomedcentral.com/1471-2288/14/43
