# Supplementary material for: Efficacy of extracellular vesicles of different cell origins in traumatic brain injury: A systematic review and network meta-analysis
Source: Front Neurosci. 2023 Mar 29;17:1147194. doi: 10.3389/fnins.2023.1147194 (PMC10090410; doi:10.3389/fnins.2023.1147194)
Supplement: Supplementary file 1 [file Data_Sheet_1.doc]

**Supplementary table 1.** **Detailed article retrieval strategy.**

| **#** | **Search query for databases (inception-2022.11.10)** | **Hits** |
| --- | --- | --- |
| **PubMed** | | |
| **1** | "brain concussion*"[MeSH Terms] OR "brain contusion*"[MeSH Terms] OR "chronic traumatic encephalopathy"[MeSH Terms] OR "brain injuries, traumatic"[MeSH Terms] OR "brain concussion*"[Title/Abstract] OR "brain contusion*"[Title/Abstract] OR "chronic traumatic encephalopathy"[Title/Abstract] OR "traumatic brain injury*"[Title/Abstract] OR "TBIs"[Title/Abstract] | **55507** |
| **2** | "extracellular vesicle*"[MeSH Terms] OR "exosome*"[MeSH Terms] OR "cell derived microparticle*"[MeSH Terms] OR "exosome*"[Title/Abstract] OR "extracellular vesicle*"[Title/Abstract] OR "nano sized vesicle*"[Title/Abstract] OR "micro vesicle*"[Title/Abstract] OR "cell derived microparticle*"[Title/Abstract] OR "shedding vesicles"[Title/Abstract] OR "apoptotic bodies"[Title/Abstract] | **41665** |
| **3** | #1 AND #2 | **219** |
| **Ovid-Embase** | | |
| **1** | ("brain concussion*" or "brain contusion*" or "chronic traumatic encephalopathy" or "brain injuries, traumatic" or "brain concussion*" or "brain contusion*" or "chronic traumatic encephalopathy" or "traumatic brain injury*" or "TBIs" or "TBI").mp. [mp=title, abstract, full text, caption text] | **70565** |
| **2** | ("extracellular vesicle*" or "exosome*" or "cell derived microparticle*" or "nano sized vesicle*" or "microvesicle*" or "shedding vesicle*" or "apoptotic bodies").mp. [mp=title, abstract, full text, caption text] | **35430** |
| **3** | #1 AND #2 | **964** |
| **The Cochrane Library** | | |
| **1** | ("brain concussion*" OR "brain contusion*" OR "chronic traumatic encephalopathy" OR "brain injuries, traumatic" OR "brain concussion*" OR "brain contusion*" OR "chronic traumatic encephalopathy" OR "traumatic brain injury*" OR "TBIs" OR "TBI"):ti,ab,kw | **3854** |
| **2** | MeSH descriptor: [Brain Injuries, Traumatic] explode all tree | **1113** |
| **3** | #1 OR #2 | **3854** |
| **4** | ("extracellular vesicle*" OR "exosome*" OR "cell derived microparticle*" OR "nano sized vesicle*" OR "microvesicle*" OR "shedding vesicle*" OR "apoptotic bodies"):ti,ab,kw | **261** |
| **5** | MeSH descriptor: [Exosomes] explode all trees | **15** |
| **6** | MeSH descriptor: [Extracellular Vesicles] explode all trees | **127** |
| **7** | #4 OR #5 OR #6 | **348** |
| **8** | #3 AND #7 | **2** |
| **Web of Science** | | |
| **1** | TS=("brain concussion*" OR "brain contusion*" OR "chronic traumatic encephalopathy" OR "brain injuries, traumatic" OR "brain concussion*" OR "brain contusion*" OR "chronic traumatic encephalopathy" OR "traumatic brain injury*" OR "TBIs" OR "TBI") | **55114** |
| **2** | TS=("extracellular vesicle*" OR "exosome*" OR "cell derived microparticle*" OR "nano sized vesicle*" OR "microvesicle*" OR "shedding vesicle*" OR "apoptotic bodies") | **45044** |
| **3** | #1 AND #2 | **294** |

**Supplementary table 2. Comparison of parameters under different models.**

|  | consistency | | | inconsistency | | |
| --- | --- | --- | --- | --- | --- | --- |
| mNSS-D1 | Dbar | pD | DIC | Dbar | pD | DIC |
|  | 25.75127 | 22.27682 | 48.02809 | 25.76234 | 22.25758 | 48.01992 |
|  | 24 data points | ratio 1.073 | I^2 = 11% | 24 data points | ratio 1.073 | I^2 = 11% |
| mNSS-D3 | Dbar | pD | DIC | Dbar | pD | DIC |
|  | 12.03292 | 11.73102 | 23.76394 | 12.0582 | 11.75215 | 23.81035 |
|  | 12 data points | ratio 1.003 | I^2 = 9% | 12 data points | ratio 1.005 | I^2 = 9% |
| mNSS-D7 | Dbar | pD | DIC | Dbar | pD | DIC |
|  | 30.67037 | 27.83789 | 58.50826 | 30.68842 | 27.84436 | 58.53278 |
|  | 30 data points | ratio 1.022 | I^2 = 5% | 30 data points | ratio 1.023 | I^2 = 6% |
| mNSS-D14 | Dbar | pD | DIC | Dbar | pD | DIC |
|  | 28.42096 | 26.87282 | 55.29378 | 28.3498 | 26.83076 | 55.18056 |
|  | 28 data points | ratio 1.015 | I^2 = 5% | 28 data points | ratio 1.012 | I^2 = 5% |
| mNSS-D21 | Dbar | pD | DIC | Dbar | pD | DIC |
|  | 20.0255 | 19.55496 | 39.58046 | 20.06211 | 19.57951 | 39.64162 |
|  | 20 data points | ratio 1.001 | I^2 = 5% | 20 data points | ratio 1.003 | I^2 = 5% |
| mNSS-D28 | Dbar | pD | DIC | Dbar | pD | DIC |
|  | 18.19038 | 17.6414 | 35.83178 | 18.2295 | 17.681 | 35.9105 |
|  | 18 data points | ratio 1.011 | I^2 = 7% | 18 data points | ratio 1.013 | I^2 = 7% |
| MWM-escape latency | Dbar | pD | DIC | Dbar | pD | DIC |
|  | 26.03397 | 24.98808 | 51.02205 | 26.05956 | 24.99057 | 51.05013 |
|  | 26 data points | ratio 1.001 | I^2 = 4% | 26 data points | ratio 1.002 | I^2 = 4% |
| MWM-time spent in the goal quadrant | Dbar | pD | DIC | Dbar | pD | DIC |
|  | 27.93781 | 27.25369 | 55.1915 | 27.95241 | 27.269 | 55.22141 |
|  | 28 data points | ratio 0.9978 | I^2 = 3% | 28 data points | ratio 0.9983 | I^2 = 3% |

**
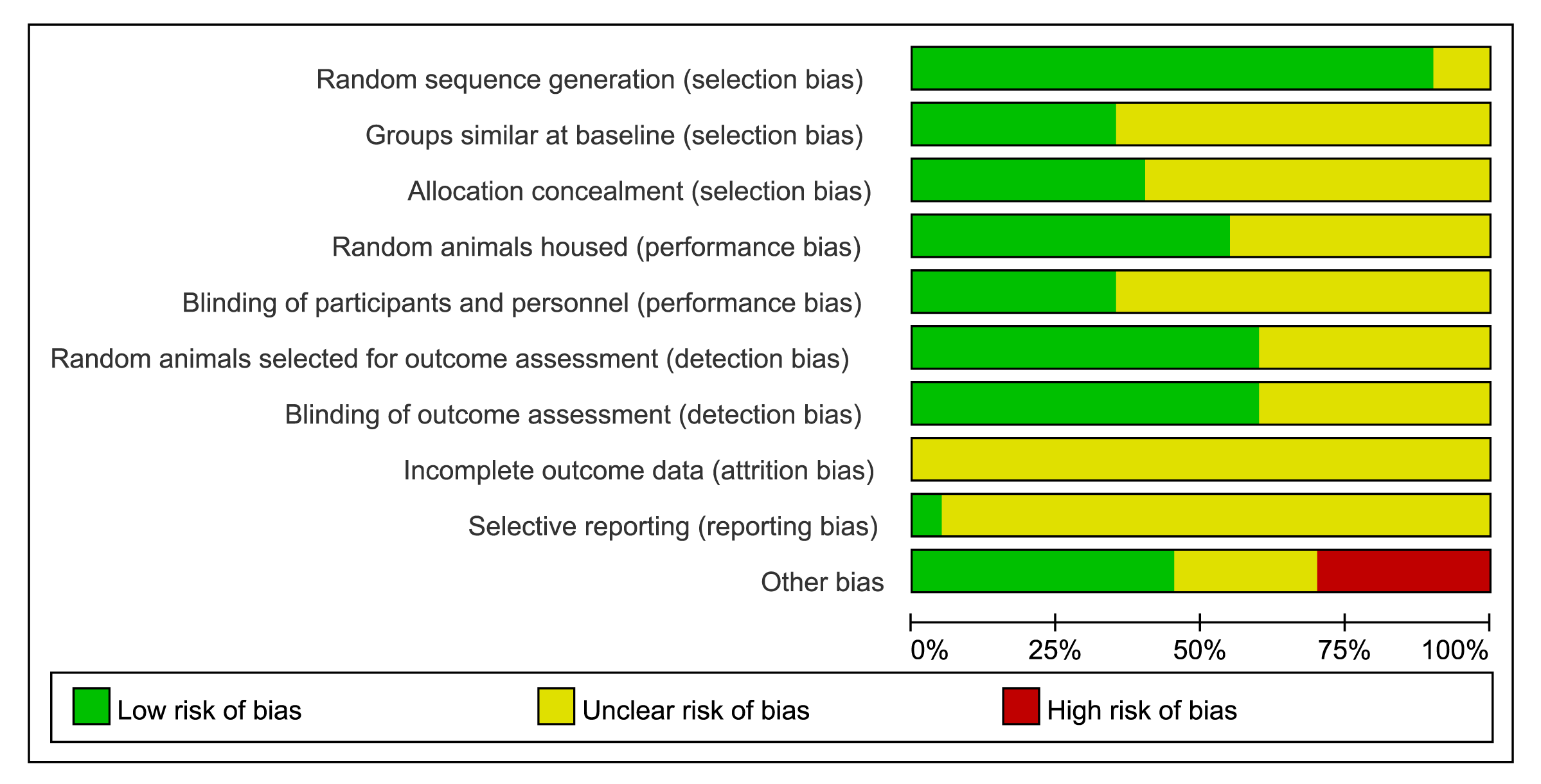
**

**Supplementary Figure 1A: Risk of bias graph with** **SYRCLE tool.**

**
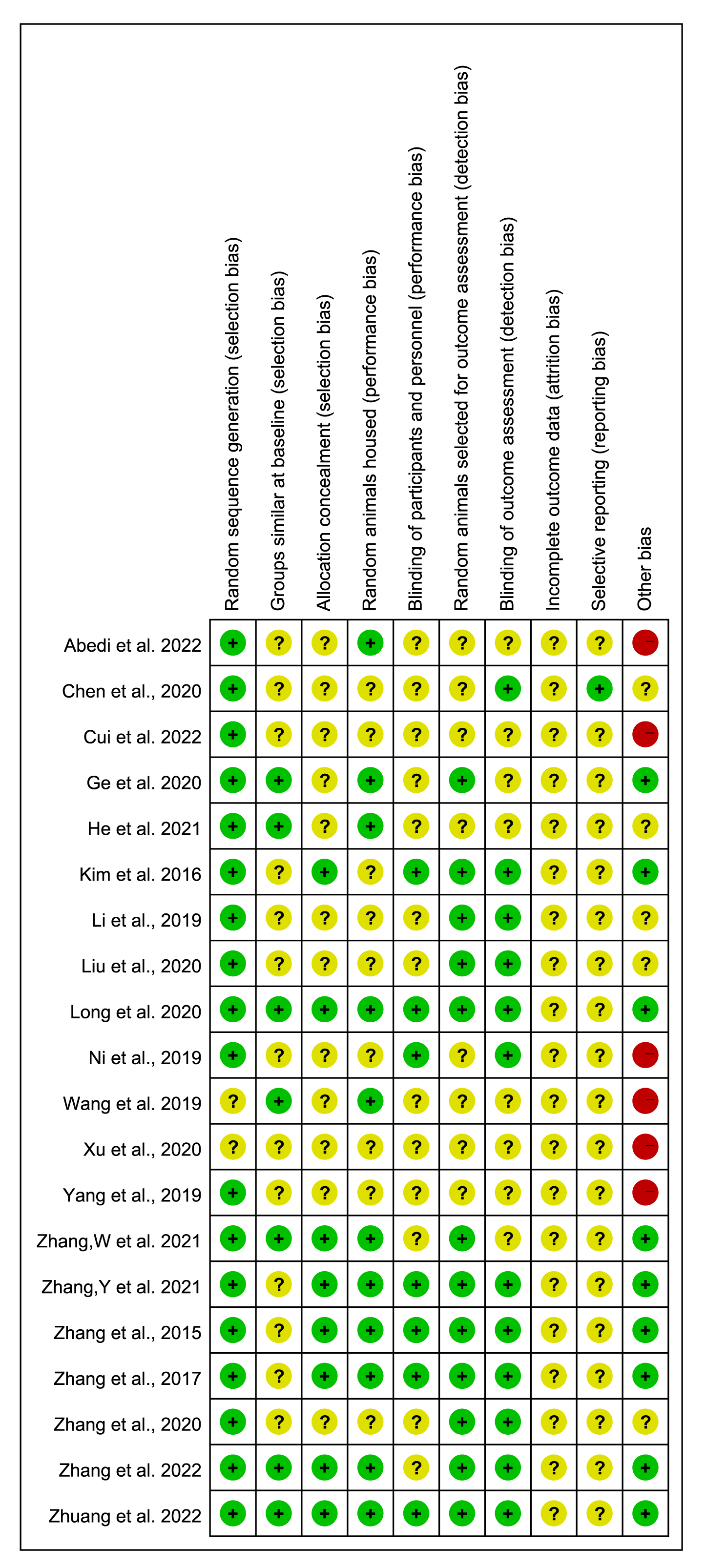
**

**Supplementary Figure 1B: Risk of bias summary with SYRCLE tool.**
